# Supplementary material for: Phenolic Metabolites from Barley in Contribution to Phenome in soil Moisture Deficit
Source: Int J Mol Sci. 2020 Aug 21;21(17):6032. doi: 10.3390/ijms21176032 (PMC7503775; doi:10.3390/ijms21176032)
Supplement: Supplementary file 1 [file ijms-21-06032-s001.zip › Table_S2 210820.docx]

**Phenolic metabolites from barley in contribution to phenome in soil moisture deficit**

**Anna Piasecka, Aneta Sawikowska, Anetta Kuczyńska, Piotr Ogrodowicz, Krzysztof Mikołajczak,**

**Paweł Krajewski, and Piotr Kachlicki**

Supplementary Table S2. Identification of phenolic metabolites which occurred only in samples of three barley varieties treated with drought regimes (present in two biological repetitions).

| No | Rt [min] | mw | ESI (-) ions  fragmentation | Compound name | Maresi 6, I | CamB1/CI 6, I | Stratus 6, I | Maresi 10, I | CamB1/CI 10, I | Stratus 10, I | Maresi 6, II | CamB1/CI 6, II | Stratus 6, II | Maresi 10, II | CamB1/CI 10, II | Stratus 10, II | Maresi 6, I+II | CamB1/CI 6, I+II | Stratus 6, I+II | Maresi 10, I+II | CamB1/CI 10, I+II | Stratus 10, I+II | references | Identification level^c^ | Databases id |
| --- | --- | --- | --- | --- | --- | --- | --- | --- | --- | --- | --- | --- | --- | --- | --- | --- | --- | --- | --- | --- | --- | --- | --- | --- | --- |
| 105 | 10.9 | 368 | 367, 193 | 3-feruloylquinic acid |  |  |  |  |  |  | * |  |  |  |  |  |  |  |  |  |  |  | Clifford et al. 2003 | 2 | CID: 9799386 |
| 106 | 11.8 | 756 | 755, 593, 473, 413. 293 | isovitexin 7*-O-*glucoside 2"*-O-*glucoside |  |  |  |  |  |  |  |  |  |  |  |  |  |  |  |  | * | * | [23] | 3 |  |
| 107 | 14.4 | 756 | 755, 593, 443, 323 | isoscoparin 7*-O-*glucoside 2"*-O-*arabinoside |  |  |  |  |  |  | * |  |  |  |  |  |  |  |  |  |  |  | [23] | 3 |  |
| 108 | 19.3 | 1124 | 1123, 961, 755, 431, 311 | sinapoyl- isovitexin 7*-O-*glucoside diglucoside |  |  |  |  | * |  |  |  |  |  |  |  |  |  |  |  |  |  | [22] | 3 |  |
| 109 | 12,9 | 726 | 725, 575, 293 | isovitexin 2"*-O-*arabinoside 6"*-O-*glucoside |  |  | * |  |  |  |  |  |  |  |  |  |  |  |  |  |  |  | [23] | 3 |  |
| 110 | 13 | 192 | 191, 111 | hydroxyferulic acid | * |  |  | * | * |  |  |  |  |  |  |  |  |  |  |  |  |  | [22] | 3 | CHEBI : 20582 |
| 111 | 15,1 | 580 | 579, 489, 399, 369 | luteolin 6*-C-*glucoside 8*-C-*arabinoside |  |  |  |  |  |  | * |  |  |  |  |  | * |  |  |  |  |  | [22] | 3 | CHEBI : **3421** |
| 112 | 15,2 | 386 | 385, 267, 249, 223, 203 | sinapic acid glucoside |  |  |  | * | * |  |  |  |  |  |  |  | * | * |  | * |  |  | [22] | 3 |  |
| 113 | 15,5 | 388 | 387, 207, 223, 247, 191, 163 | sinapic acid derivative |  |  |  | * |  |  |  |  |  |  |  |  |  |  |  | * |  |  | [22] | 3 | ` |
| 114 | 17 | 352 | 351, 249, 223 | sinapic acid derivative | * |  |  | * | * |  |  |  |  |  |  |  |  | * |  | * |  |  | [22] | 3 |  |
| 115 | 23,4 | 756 | 755, 413, 293 | isovitexin 2"*-O-*diglucoside |  |  |  |  |  |  |  |  |  |  | * |  |  |  |  |  |  |  | [23] | 3 |  |
| 116 | 18,9 | 726 | 725, 447, 327 | isoorientin 7*-O-*rhamnosylarabinoside |  |  |  |  |  |  |  |  |  | * |  |  |  |  |  |  |  |  | [22] | 3 |  |
| 117 | 19,1 | 742 | 741, 595, 301 | quercetin *O-*rhamnoside *O-*arabinosylglucoside |  | * |  |  |  |  |  |  |  |  |  |  |  |  |  |  |  |  | [22] | 3 |  |
| 118 | 16,1 | 918 | 917, 755, 447, 357, 327 | isoorientin 7*-O-*rhamnosylglucoside 4'*-O-*glucoside |  |  |  |  |  |  |  |  |  |  |  |  |  | * |  | * |  |  | [22] | 3 |  |
| 119 | 18,5 | 726 | 725, 579, 285 | luteolin *O-*rhamnoside *O-*arabinosylglucoside |  | * |  |  |  |  |  |  |  |  |  |  |  |  |  |  |  |  | [22] | 3 |  |
| 120 | 20 | 594 | 593, 473, 429, 309 | isoorientin 2"*-O-*rhamnoside |  |  |  |  |  |  | * |  |  |  |  |  |  |  |  |  |  |  | [23] | 2 | CHEBI : 28596 |
| 121 | 20,5 | 580 | 579, 459, 429, 357, 327 | isoorientin 2"*-O-*arabinoside |  |  |  |  |  |  | * |  |  | * |  |  |  |  |  |  |  |  | [23] | 2 | CID: 44257956 |
| 122 | 21,5 | 788 | 787, 431, 311 | isovitexin derivative |  |  |  |  |  | * |  |  |  |  |  |  |  |  |  |  |  |  | [23] | 3 |  |
| 123 | 21,6 | 520 | 519, 325, 223, 164 | sinapic acid derivative |  |  |  | * |  |  |  |  |  |  |  |  |  | * |  | * |  |  | [22] | 3 |  |
| 124 | 23,3 | 578 | 577, 413, 293 | isovitexin 2"*-O-*rhamnoside |  |  |  |  |  |  | * |  |  |  |  |  | * |  |  |  |  |  | [23] | 2 | CID: 44257672 |
| 125 | 24,5 | 740 | 739, 431, 311 | isovitexin 7*-O-*[6"-*p*-coumaroyl]-glucoside |  |  |  |  |  |  |  |  |  |  |  | * | * |  |  |  |  |  | [23] | 3 | CID: 44257783 |
| 126 | 24,6 | 504 | 503, 223 | sinapic acid derivative |  |  |  |  | * |  |  |  |  |  |  |  |  |  |  |  |  |  | [22] | 3 |  |
| 127 | 25,1 | 738 | 737, 657, 455, 329 | tricin derivative |  |  | * |  |  |  |  |  |  |  |  |  |  |  |  |  |  |  | [24] | 3 |  |
| 128 | 25,6 | 768 | 767, 687, 525, 329 | tricin derivative |  |  | * |  |  |  |  |  |  |  |  |  |  |  |  |  |  |  | [24] | 3 |  |
| 129 | 25,9 | 978 | 977, 815, 461, 341 | isoscoparin 7*-O-*[6''-hydroxyferuloyl] -glucoside 4'*-O-*glucoside |  |  |  |  | * | * | * |  |  |  |  |  |  |  |  |  |  |  | [23] | 3 |  |
| 130 | 26,2 | 962 | 961, 799, 593, 431, 311 | isovitexin 7*-O-*[6''-sinapoyl]-glucoside 4'*-O-*glucoside |  |  |  |  |  |  |  |  |  |  |  |  | * |  |  |  |  |  | [23] | 3 | CHEBI : **75564** |
| 131 | 26,8 | 948 | 947, 785, 431, 311 | isovitexin 7*-O-*[6"-hydroxyferuloyl]-glucoside 4’*-O-*glucoside |  |  |  |  |  |  |  |  |  |  |  |  |  | * |  |  |  |  | [23] | 3 |  |
| 132 | 27,4 | 432 | 431, 269 | apigenin 7*-O-*glucoside |  |  |  |  |  | * |  |  |  |  |  |  |  |  |  |  |  |  | std | 1 | CHEBI : **16778** |
| 133 | 27,8 | 786 | 785, 593, 413, 293 | hydroxyferuloyl-isovitexin 2"*-O-*glucoside |  |  |  |  |  |  |  |  |  |  |  |  |  |  |  | * |  |  | [22] | 3 |  |
| 134 | 28,3 | 932 | 931, 769, 593, 413, 293 | feruloyl-isovitexin O-glucoside 2"*-O-*glucoside |  |  |  |  |  |  | * |  |  |  |  |  |  |  |  |  |  |  | [22] | 3 |  |
| 135 | 28,5 | 654 | 653, 329 | tricin 7*-O-*[6"-caffeoyl]-glucoside |  |  |  |  | * |  |  |  |  |  |  |  |  |  |  |  |  |  | [24] | 3 |  |
| 136 | 28,9 | 932 | 931, 769, 563, 413, 293 | isovitexin 7*-O-*[6''-sinapoyl]-glucoside 2''*-O-*arabinoside | * |  |  |  | * |  |  |  |  | * |  |  |  |  |  | * |  |  | [23] | 3 |  |
| 137 | 29,8 | 740 | 739, 563, 413, 293 | feruloyl- isovitexin 2''-*O*-arabinoside |  |  |  | * |  |  |  |  |  |  |  |  | * |  |  |  |  |  | [22] | 3 |  |
| 138 | 30,8 | 800 | 799, 593, 431, 311 | sinapoyl-isovitexin 7*-O-*glucoside |  |  |  |  |  |  |  | * |  |  |  |  |  |  |  |  | * | * | [22] | 3 |  |
| 139 | 30,9 | 608 | 607, 283, 269 | apigenin 7*-O-*[6"-feruloyl]-glucoside |  |  |  | * | * |  |  |  |  |  |  |  |  |  |  |  |  |  | [24] | 3 |  |
| 140 | 32 | 816 | 815, 695, 623, 489, 327 | sinapoyl-isoorientin glucoside |  |  |  |  |  |  | * |  |  |  |  |  |  |  |  |  |  |  | [22] | 3 |  |
| 141 | 33,5 | 800 | 799, 623, 443, 323 | feruloyl-isoscoparin 2"*-O-*glucoside |  |  |  |  |  |  | * |  |  |  |  |  |  |  |  |  |  |  | [22] | 3 |  |
| 142 | 34,9 | 860 | 859, 697, 529, 329 | tricin 7*-O-*[sinapoyl]-glucoside 4'*-O-*glucoside |  |  |  | * |  |  |  |  |  |  |  |  |  |  |  |  |  |  | [24] | 3 | CHEBI : 75461 |
| 143 | 34,8 | 592 | 591, 299, 284 | chrysoeriol 7*-O-*dirhamnoside |  |  |  | * |  |  |  |  |  |  |  |  |  |  |  |  |  |  | [21] | 3 |  |
| 144 | 36,5 | 830 | 829, 697, 623, 329 | sinapoyl-tricin arabinosylglucoside |  |  |  |  |  |  |  |  |  |  |  |  |  | * |  | * |  |  | [21] | 3 |  |
| 145 | 36,8 | 948 | 947, 929, 605, 413, 293 | hydroxyferuloyl-isovitexin 2''*-O-*diglucoside |  |  |  | * |  |  |  |  |  |  |  |  |  |  |  |  |  |  | [22] | 3 |  |
| 146 | 37 | 962 | 961, 769, 593, 413, 299 | feruloyl-hydroxyferuloyl-isovitexin 2''*-O-*glucoside |  |  |  |  |  |  |  |  |  |  |  |  |  |  |  | * |  |  | [23] | 3 |  |
| 147 | 37,9 | 1006 | 1005, 799, 781, 619, 593, 473, 413, 293 | disinapoyl-isovitexin 2''*-O-*glucoside |  |  |  |  |  |  |  |  |  |  |  |  |  |  |  | * |  |  | [22] | 3 |  |
| 148 | 38 | 948 | 947, 593, 489, 431, 311 | isovitexin 7*-O-*glucoside 4'*-O-*[6"-hydroxyferuloyl]-glucoside |  |  |  |  |  |  |  |  |  |  | * |  |  |  |  |  |  |  | [23] | 3 |  |
| 149 | 38,5 | 638 | 637, 329 | tricin rhamnosylglucoside |  |  |  |  |  |  | * |  |  |  |  |  |  |  |  |  | * | * | [22] | 3 |  |
| 150 | 39,1 | 624 | 623, 329, 314 | tricin arabinosylglucoside |  |  |  |  |  |  | * |  |  |  |  |  |  |  |  |  |  |  | [22] | 3 |  |
| 151 | 39,7 | 448 | 447, 327 | isoorientin |  |  |  |  | * |  |  |  |  |  |  |  |  |  |  |  |  |  | std | 1 | CHEBI : 17965 |
| 152 | 40,3 | 518 | 517, 473, 311 | isovitexin malonylated |  |  |  |  | * |  |  |  |  |  |  |  |  |  |  |  |  |  | [22] | 3 |  |
| 153 | 42 | 520 | 519, 475, 355, 271 | naringenin 7*-O-*glucoside malonylated |  |  |  |  | * |  |  |  |  |  |  |  |  |  |  |  |  |  | [22] | 3 |  |

* - presence of metabolites in samples

6, I - 6th day of drought I; 10, I - 10th day of drought I; 6, II - 6th day of drought II; 10, II - 10th day of drought II; 6, I+II - 6th day of drought I+II; 10, I+II - 10th day of drought I+II

**^c^** -metabolite identification level according to Metabolomics Standards Initiative recommendation (Sumner et al. 2007)

databases id - identifiers for a chemical structure in online available databases: CHEBI - the CHEBI database and ontology (Chemical Entities of Biological Interest), CID - the PubChem Compound database

Clifford et al. 2003 is reffered to Clifford, M.N.; Johnston, K.L.; Knight, S.; Kuhnert, N. Hierarchical scheme for LC-MSn identification of chlorogenic acids. *J. Agric. Food Chem*. **2003**, *51*, 2900.
