# Supplementary material for: Phenolic Metabolites from Barley in Contribution to Phenome in soil Moisture Deficit
Source: Int J Mol Sci. 2020 Aug 21;21(17):6032. doi: 10.3390/ijms21176032 (PMC7503775; doi:10.3390/ijms21176032)
Supplement: Supplementary file 1 [file ijms-21-06032-s001.zip › Table_S4 100820.docx]

**Phenolic metabolites from barley in contribution to phenome in soil moisture deficit**

**Anna Piasecka, Aneta Sawikowska, Anetta Kuczyńska, Piotr Ogrodowicz, Krzysztof Mikołajczak,**

**Paweł Krajewski, and Piotr Kachlicki**

Supplementary Table S4. Pearson correlation coefficients between variety-specific effects of treatment I on phenotypic and metabolomic traits significant at P < 0.05.. Correlations marked in gray are significant at P < 0.01. Blank cells indicate no significant correlation.

| Traits | | Metabolites | | | | | | | | |
| --- | --- | --- | --- | --- | --- | --- | --- | --- | --- | --- |
|  |  | M8 | M44 | M48 | M54 | M82 | M84 | M92 | M99 | M104 |
| Phenotypic traits | F1 |  |  |  |  |  |  |  |  | 0.66 |
|  | F2 | 0.64 | 0.77 | 0.74 |  |  |  |  | 0.63 |  |
|  | F3 |  | 0.80 | 0.67 |  |  |  |  |  |  |
|  | F7 |  | -0.70 |  |  |  |  |  |  |  |
|  | F10 |  |  |  |  |  |  |  |  |  |
|  | F13 | -0.76 | -0.75 | -0.77 | -0.89 | -0.81 | -0.71 |  | -0.76 |  |
|  | F14 | -0.64 | -0.77 | -0.68 | -0.73 | -0.74 |  | -0.70 | -0.76 |  |
|  | F15 | -0.65 | -0.94 | -0.70 | -0.69 | -0.70 | -0.63 | -0.73 | -0.75 |  |
